# Transplantation of purified iPSC-derived cardiomyocytes in myocardial infarction

**DOI:** 10.1371/journal.pone.0173222

**Published:** 2017-05-11

**Authors:** Sebastian V. Rojas, George Kensah, Alexander Rotaermel, Hassina Baraki, Ingo Kutschka, Robert Zweigerdt, Ulrich Martin, Axel Haverich, Ina Gruh, Andreas Martens

**Affiliations:** 1 Leibniz Research Laboratories for Biotechnology and Artificial Organs (LEBAO), Hannover Medical School, Hannover, Germany; 2 Department of Cardiothoracic, Transplantation, and Vascular Surgery, Hannover Medical School, Hannover, Germany; 3 REBIRTH-Cluster of Excellence, Hannover Medical School, Hannover, Germany; University of Kansas Medical Center, UNITED STATES

## Abstract

**Background:**

Induced pluripotent stem cells (iPSC) can be differentiated into cardiomyocytes and represent a possible autologous cell source for myocardial repair. We analyzed the engraftment and functional effects of murine iPSC-derived cardiomyocytes (iPSC-CMs) in a murine model of myocardial infarction.

**Methods and results:**

To maximize cardiomyocyte yield and purity a genetic purification protocol was applied. Murine iPSCs were genetically modified to express a Zeocin^™^ resistance gene under control of the cardiac-specific α-myosin heavy chain (α-MHC, MYH6) promoter. Thus, CM selection was performed during in vitro differentiation. iPSC-CM aggregates (“cardiac bodies”, CBs) were transplanted on day 14 after LAD ligation into the hearts of previously LAD-ligated mice (800 CBs/animal; 2-3x10^6^ CMs). Animals were treated with placebo (PBS, n = 14) or iPSC-CMs (n = 35). Myocardial remodeling and function were evaluated by magnetic resonance imaging (MRI), conductance catheter (CC) analysis and histological morphometry. *In vitro* and *in vivo* differentiation was investigated. Follow up was 28 days (including histological assessment and functional analysis). iPSC-CM purity was >99%. Transplanted iPSC-CMs formed mature grafts within the myocardium, expressed cardiac markers and exhibited sarcomeric structures. Intramyocardial transplantation of iPSC-CMs significantly improved myocardial remodeling and left ventricular function 28 days after LAD-ligation.

**Conclusions:**

We conclude that iPSCs can effectively be differentiated into cardiomyocytes and genetically enriched to high purity. iPSC derived cardiomyocytes engraft within the myocardium of LAD-ligated mice and contribute to improve left ventricular function.

## Introduction

Cardiovascular diseases represent the most important burden of the present century with increasing numbers of afflicted patients worldwide [1]. Once damaged by myocardial infarction, the hearts limited ability of self-regeneration often culminates in irreversible congestive heart failure (CHF) [2]. Advances in medical therapy have improved the outcome in these patients, however once reached it’s end stage, CHF can only be treated by cardiac transplantation or ventricular assist devices (VAD) [3–8]. With the purpose of finding an alternative treatment, capable to regenerate infarcted myocardium, a manifold of studies has evaluated stem cells in preclinical and clinical trials [9–20]. Next to important factor like biodistrution, retention and graft viability [19–21], one of the main challenges in this field is to find the right cell source as there is a wide assortment of different stem cell types: adult cardiac progenitor-, bone marrow- (BMSCs), embryonic- (ESCs) and lately, induced pluripotent stem cells (iPSCs) [22]. Moderate success of other stem cell types and the unique capability of iPSCs to differentiate into de novo cardiomyocytes (CMs) have raised expectations about this regenerative source [23–26]. In other words, the formation of mature *de novo* myocardium *in vivo* may be best achieved by using iPSC-derived cardiomyocytes (CMs). However, the process of harvesting CMs from iPSCs faces several hurdles: Standard protocols are based on spontaneous differentiation or directed differentiation of pluripotent stem cells (PSCs) being hampered by high cell heterogenicity and limited cardiomyocyte maturation with poor purity [27]. Furthermore, selection protocols should be able to eliminate undifferentiated iPSCs or highly proliferative progenitors that might form teratomas *in vivo* [28,29]. To obtain reasonable amounts of cells for transplantation purposes the upscaling of culture conditions is also needed [30,31]. Finally, the viability of dissociated CMs should be improved [32–34].

Addressing these challenges, our group has recently reported a novel method for efficient cardiac differentiation followed by a genetic purification method to produce high numbers of ultrapure (>99%) CMs from murine and human iPSCs [35]. The purpose of the present study was to investigate the ability of highly purified iPSC derived CMs to form mature cardiac grafts *in vivo* and to engraft after transplantion intramyocardial transplantation in an acute myocardial infarction model in mice.

## Materials and methods

### iPSC culture and genetic purification of murine iPSC-derived cardiomyocytes

Genetic cardiomyocyte purification was established by Kensah et al. [35] and is detailed in S1 File. In short, iPSCs derived from Oct4-eGFP expressing OG2 mice [36] were genetically modified to express a Zeocin^™^ resistance gene under control of the cardiac-specific α-MHC (MYH6) promoter. Cardiac differentiation was initiated by hanging drop technique. On differentiation day (dd) 3, embryoid bodies were transferred into dynamic suspension culture. Differentiation medium was supplemented with 400 μg/mL Zeocin^™^ from dd7 to dd14 to initiate cardiomyocyte (CM) selection. Resulting CM enriched aggregates (“cardiac bodies”, CBs) were characterized by immunostaining. Undifferentiated, non-selected iPSCs served as controls. Before transplantation, CBs were marked for histological detection with a vitality sensitive fluorescence marker (Vybrant^®^ CFDA SE [carboxy-fluorescein diacetate succinimidyl ester] Cell Tracer Kit, LifeTechnologies^™^, Darmstadt, Germany) as described in the Supporting Information.

### Animal care

Surgery and animal care were provided following the *Guide for the Care and Use of Laboratory Animals* (National Institutes of Health, volume 25, no 28, revised 1996) and in accordance with federal regulations. The study protocol was approved by state authorities (Niedersächsisches Amt für Verbraucherschutz und Lebensmittelsicherheit). Inhalative anesthesia with 2.5% vaporized isoflurane (Abbott, Germany) was used in all experiments. Animals received prophylactic oral antibiotic and analgesic drugs and kept under special care in the central animal laboratory of our institution. Monitoring of the animals included daily visits.

### Myocardial infarction model

A total of 70 immunodeficient SCID beige mice (15–21 g, Charles River, Germany) were used. Myocardial infarction (MI) was induced as described in the Supporting Information and performed as previously described [37]. Aliquots of 15 μL cell suspension containing 800 CBs (~2-3x10^6^ viable iPSC-CMs) in phosphate buffered saline (PBS) or PBS alone were injected into the anterior left ventricle of LAD ligated mice shortly after MI induction. Animals were divided into a sham-operated group (Sham; n = 10), a placebo treated infarct group (PBS, n = 15) and three infarct groups treated with iPSC-derived CMs (iPSC-CM^7^, n = 14, follow up 7 days, graft and infarct morphology assessment; iPSC-CM^17^, n = 3, follow up 17 days, graft assessment; iPSC-CM^28^, n = 28, follow up 28 days, complete functional and histological analysis).

### Magnetic resonance imaging

A 7 Tesla scanning system (PharmaScan, Bruker, Etlingen, Germany) was used for magnetic resonance imaging (MRI) as detailed in the Supporting Information. On postoperative day 2 (POD 2) infarct size was determined by contrast enhanced MRI. Cardiac function was evaluated on POD 27.

### Conductance catheter analysis

On POD 28 conductance catheter (CC) analysis was performed to record LV pressure-volume loops as described recently [38] and detailed in the Supporting Information. Following the operation animals were sacrificed for histological evaluation.

### Histology and immunostaining

Hearts were processed in standard fashion and histological morphometry as well as immunostaining was performed as described in the Supporting Information. iPSC-CM grafts were detected by their cell tracer staining. Graft size was measured using a pixel-based approach. Data were obtained by computer-assisted morphometry (Image J 1.40g, NIH, USA). An overview of all used antibodies for immunostaining is described in Table A in S1 File.

## Statistics

GraphPad Prism 6.01 was used for statistical analysis. If not stated otherwise, data are given as mean±SEM. Differences in mortality were analysed by Fisher’s exact test. Comparison of continuous variables was performed with Student’s T-test or one-way ANOVA followed by Tukey's multiple comparison test. Linear regression analysis was performed to correlate continuous data. Differences were considered significant at *P*<0.05. All reported *P* values are two-sided.

## Results

### Genetic selection results in aggregates with high purity of iPSC-derived functional cardiomyocytes (“cardiac bodies”)

**Fig 1:**
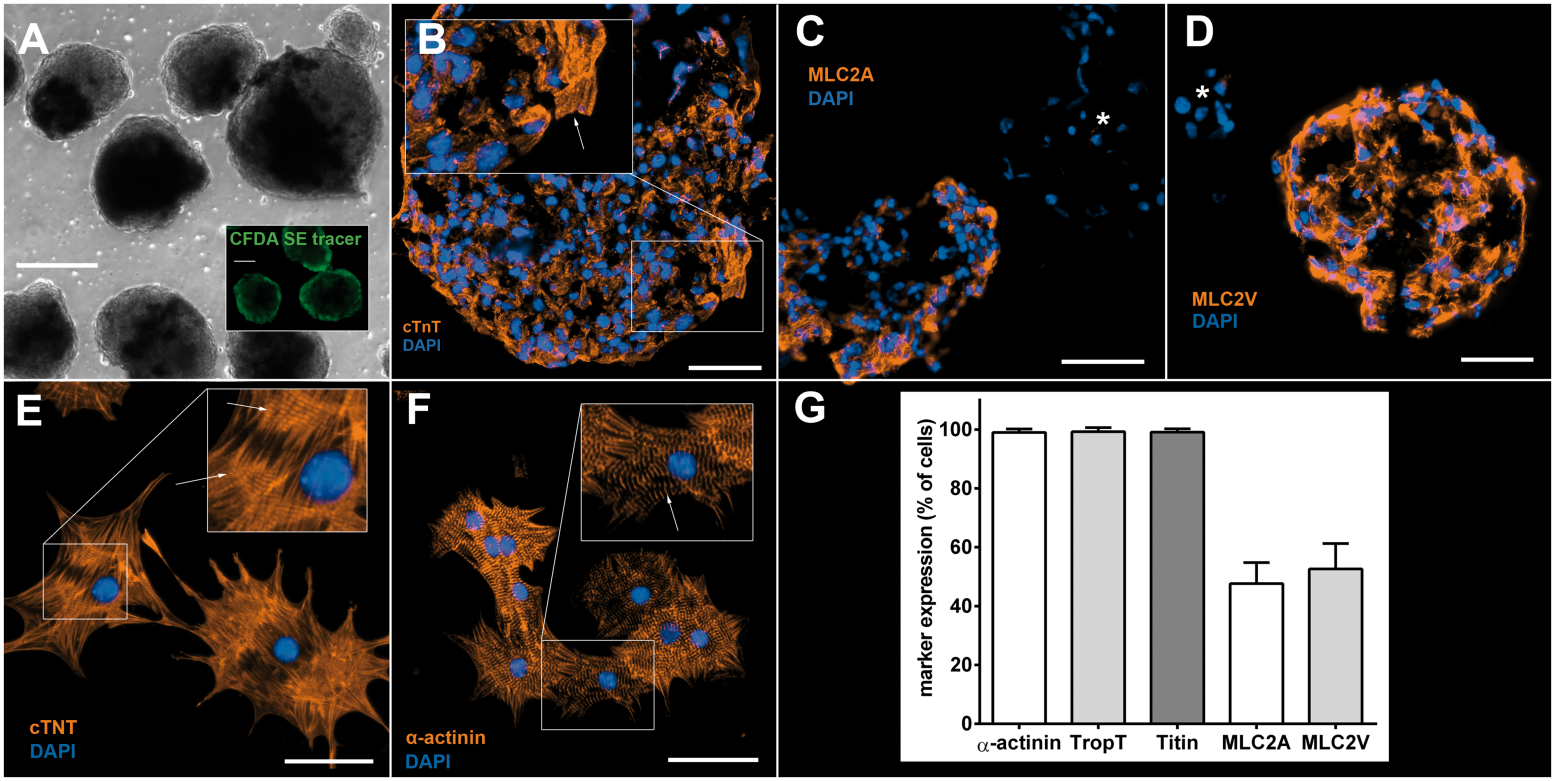
Cardiac bodies and cardiomyocytes derived from murine iPSCs. **A:** CBs after antibiotic selection (dd14, brightfield view); inset: CBs after CFDA SE tracer staining (dd14). Scale bars: 100μm. **B:** CBs are positive for cTNT and show CMs with sarcomeric striations (inset, arrow). Scale bar: 50μm. **C+D:** CBs at dd14 are positive either for MLC2A or MLC2V indicating spontaneous differentiation into both an atrial and ventricular phenotype; negative CBs are marked with *, respectively. Scale bars: 50μm. **E+F:** Reseeded CMs exhibit a mature sarcomeric intracellular organisation. Staining for cTnT and α-sarcomeric actinin shows Z-lines (arrows). Scale bars: 50μm. **G:** Relative amount of reseeded CMs expressing cardiac markers α-sarcomeric actinin (99.1±1.5%), cTnT (99.3±0.4%), Titin (99.4±0.3%), MLC2A (47.3±1.9%) and MLC2V (52.1±1.8%); N = 8.

Following cardiac differentiation and antibiotic selection with Zeocin^®^ under dynamic suspension culture conditions, murine iPSCs formed spontaneously beating aggregates of almost pure cardiomyocytes (Fig 1 and S1 Video). These aggregates were termed “cardiac bodies” (CBs) as proposed by Kensah at al. [35] and contained approximately 1500–2500 CMs with a CB size of 100–200 μm on dd14. From 9.6 x 10^6^ undifferentiated iPSCs initially inoculated we were able to retrieve an average of 3.2 x 10^6^ total CMs after differentiation and selection resulting in a ratio of 1:3 CM:undifferentiated iPSC after 14 days. In comparison to undifferentiated non-selected EBs (S1 Fig), CBs lost the Oct3/4-mediated GFP signal (S2 Fig). 4.3±3.0% of dissociated and reseeded CMs (N = 7) showed Ki-67 positive nuclei (S3 Fig). They consisted of 99.3±0.5% cardiac Troponin T (cTnT) positive cells. They were also positive for cardiac markers Titin, α-sarcomeric actinin, myosin light chain 2V (MLC2V) and MLC2A and exhibited a distinctive cross striation pattern in CBs as well as after reseeding (Fig 1B, 1E and 1F). MLC2V and MLC2A expressing CBs and reseeded CMs were almost equally distributed (Fig 1C, 1D and 1G). CBs on dd14 were positive for Connexin 45 and 40 (S4 and S5 Figs).

### CFDA SE tracer staining shows vital CMs after antibiotic selection and enables easy identification of iPSC-derived CMs *in vivo*

**Fig 2:**
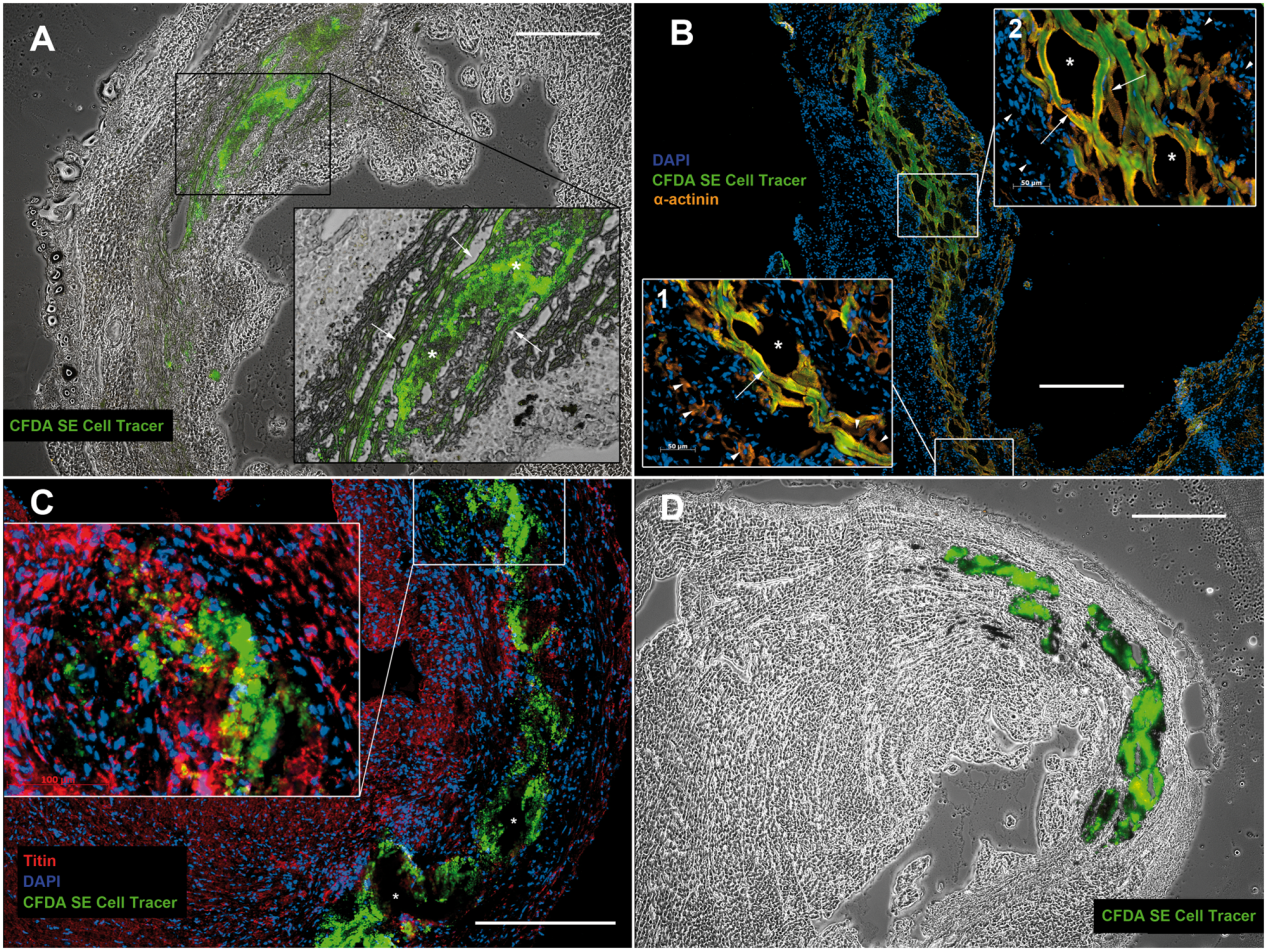
Genetically purified iPSC-derived CMs form mature grafts *in vivo*. **A+B:** CFDA SE cell tracer positive iPSC-CM grafts 7 days after intramyocardial transplantation: Adjacent to the host myocardium iPSC-CMs align in a parallel, longitudinal fashion and exhibit sarcomeric structures (arrows). Within central portions of broader grafts (approximately > 200 μm) they maintain a small, round shape (* in A). In the infarct penumbra iPSC-CMs lie in close proximity to host CMs (arrowheads in B1), occasionally with direct cell contact (arrowheads in the bottom right corner of B1). Inside the infarct area iPSC-CMs are typically surrounded by infiltrating host cells (arrowheads in B2). Tissue disruption during histological preparation (* in B1+2) indicates loose cell adhesion within iPSC-CM grafts. **C+D:** CFDA SE cell tracer positive iPSC-CM graft 28 days after intramyocardial transplantation: The cell tracer remains visible 28 days after engraftment, but iPSC-CMs develop an amorphic appearance. Sarcomeric structures are not observed. Vacuoles form during histological preparation (* in C). A+D: brightfield overlay. Scale bars: 400μm.

Using the CFDA SE tracer staining, CMs were effectively marked *in vitro* ensuring fluorophore accumulation in vital cells only (Fig 1A, S2 Video). CBs continued to contract after staining (S2 Video). For transplantation, 800 labeled CBs (2-3x10^6^ vital CMs) per animal were used. iPSC-CM grafts were readily visible within the host myocardium (Fig 2).

### Mortality

Overall mortality was 0% in the Sham group, 33% in the PBS group, 25% in the iPSC-CM^7^ group and 25% in the iPSC-CM^28^ group, respectively (S6 Fig).

### Purified iPSC-derived CMs form large intramyocardial grafts exhibiting mature cardiac features

Seven days after intramyocardial transplantation iPSC-derived CMs formed large graft bands within the infarct region as well as the adjacent non-infarcted myocardium. Grafted cells exhibited a longitudinal alignment parallel with the LV wall (Fig 2A and 2B). They were typically separated from viable host myocardium within the infarcted area by infiltrating cells (Fig 2B). Transplanted iPSC-derived CMs developed a typical CM-like morphology *in vivo* (Fig 2B). They expressed cardiac markers and showed mature sarcomeric structures as identified by a distinctive cross striation pattern (Fig 2B) up to 17 days after transplantation. Cryoconservation and–sectioning resulted in a localized tissue disruption within CM grafts (Fig 2B).

**Fig 3:**
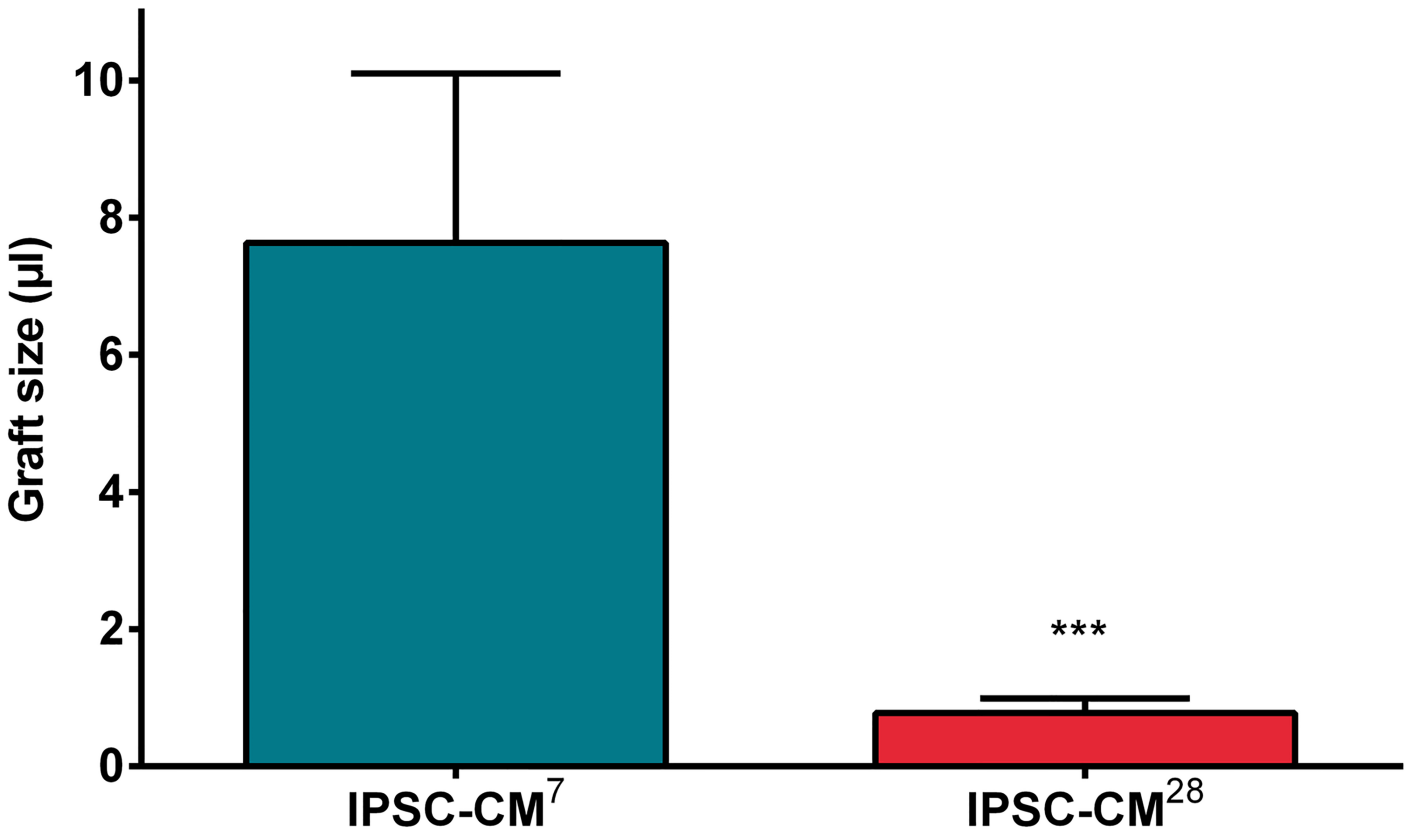
Graftsize after intramyocardial transplantation of iPSC derived CMs. Intramyocardial grafts were detected by their CFDA SE fluorescence on POD7 and POD28. Graft size (μl): after 7 days: 7.6±2.5; after 28 days: 0.78±0.21. *** *P*<0.001.

Graft size significantly decreased between 7 and 28 days after transplantation (Fig 3). Although iPSC-CM grafts could be well identified within the myocardium after 28 days, they developed an amorphic appearance and showed vacuoles after tissue preparation (Fig 2C and 2D) indicating cell death. Sarcomeric structures could not be observed 28 days after transplantation (Fig 2C).

### Intramyocardial transplantation of purified iPSC-derived CMs improves ventricular remodeling and function

LAD ligation resulted in large myocardial infarcts of 36±14% of LV mass as determined by contrast enhanced MRI on day 2 post infarction. The ischemic area at risk did not differ in size between the groups. Progressive myocardial remodeling was observed in infarcted animals over a course of 28 days as determined by MRI, CC analysis and morphometry.

#### MRI and CC analysis

**Fig 4:**
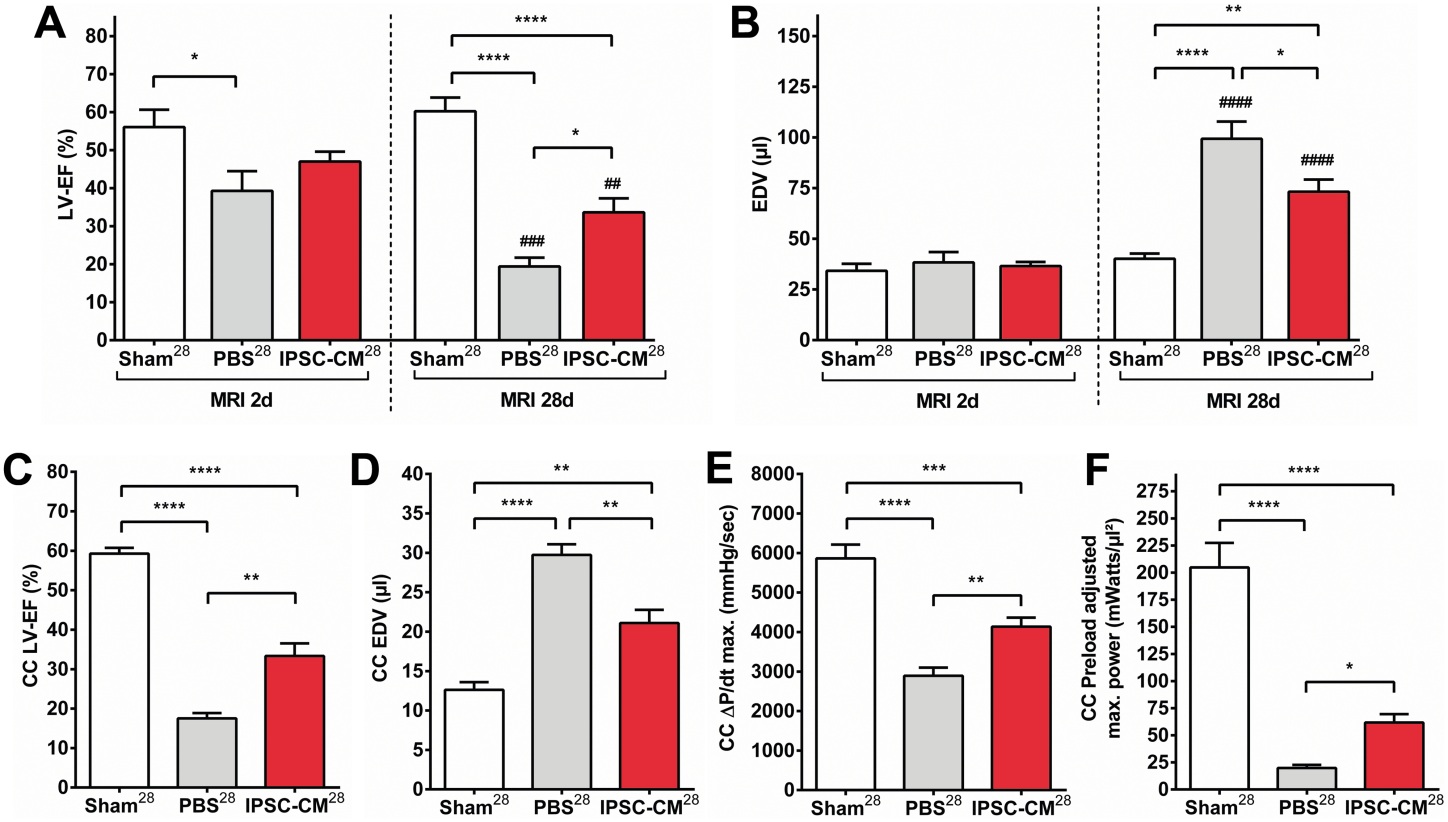
Intramyocardial transplantation of iPSC derived CMs improves ventricular remodeling and function after myocardial infarction. Hemodynamic evaluation by magnetic resonance imaging (MRI; POD 27; A+B) and conductance catheter analysis (CC; POD 28; C-F). **A**: Left ventricular ejection fraction (LV-EF [%]) as measured by MRI: On POD 2: Sham^28^ = 56±5; PBS^28^ = 39±5; iPSC-CM^28^ = 47±3. On POD 27: Sham^28^ = 60±4; PBS^28^ = 19±2; iPSC-CM^28^ = 34±4 **B:** End-diastolic volume (EDV [μl]) as measured by MRI: On POD 2: Sham^28^ = 34±3; PBS^28^ = 38±5; iPSC-CM^28^ = 37±2. On POD 27: Sham^28^ = 40±3; PBS^28^ = 99±9; iPSC-CM^28^ = 73±6 **C:** LV-EF (%) as measured by CC on POD 28: Sham^28^ = 59±1; PBS^28^ = 18±1; iPSC-CM^28^ = 33±3 **D:** End-diastolic volume (EDV [μl]) as measured by CC on POD 28: Sham^28^ = 13±1; PBS^28^ = 30±1; iPSC-CM^28^ = 21±2 **E:** maximum Pressure increase (ΔP/dt max. [mmHg/sec]) as measured by CC on POD 28: Sham^28^ = 5863±351; PBS^28^ = 2893±207; iPSC-CM^28^ = 4135±232 **F:** Preload adjusted maximal power (mWatts/μl^2^) as measured by CC on POD 28: Sham^28^ = 205±23; PBS^28^ = 20±3; iPSC-CM^28^ = 62±8. *P<0.05; **P<0.01; ***P<0.001; ****P<0.0001 (for group comparison); ##*P*<0.01; ###*P*<0.001; ####*P*<0.0001 (for paired longitudinal comparison).

LAD-ligation resulted in a marked reduction of LV function and in a volume overload compared to sham operated animals after 28 days (Fig 4A and 4B). Whereas end diastolic volume (EDV) was comparable between infarcted and non-infarcted animals 2 days post MI, myocardial remodeling led to LV enlargement in infarcted hearts after 28 days with an average 2.0-fold increase in EDV (Fig 4B). Myocardial remodeling was significantly improved in iPSC-CM treated animals compared to infarcted controls as demonstrated by a lesser degree of volume overload and dilatation (Fig 4B). This correlated with a significantly higher LV ejection fraction (LV-EF) in the iPSC-CM^28^ group (Fig 4A). MRI findings correlated well with CC measurements (S7 Fig), volume values were typically underestimated by CC evaluation. Based on the MRI data, the relative improvement of LV-EF compared to PBS treated controls 172% for iPSC-CM treated animals. Additional evaluation of myocardial contractility by CC analysis revealed a significantly improved maximum pressure increase (dP/dt max) as well as a significantly higher preload adjusted maximum power in the iPSC-CM group compared to infarcted controls (Fig 4E and 4F).

#### Morphometry

**Fig 5:**
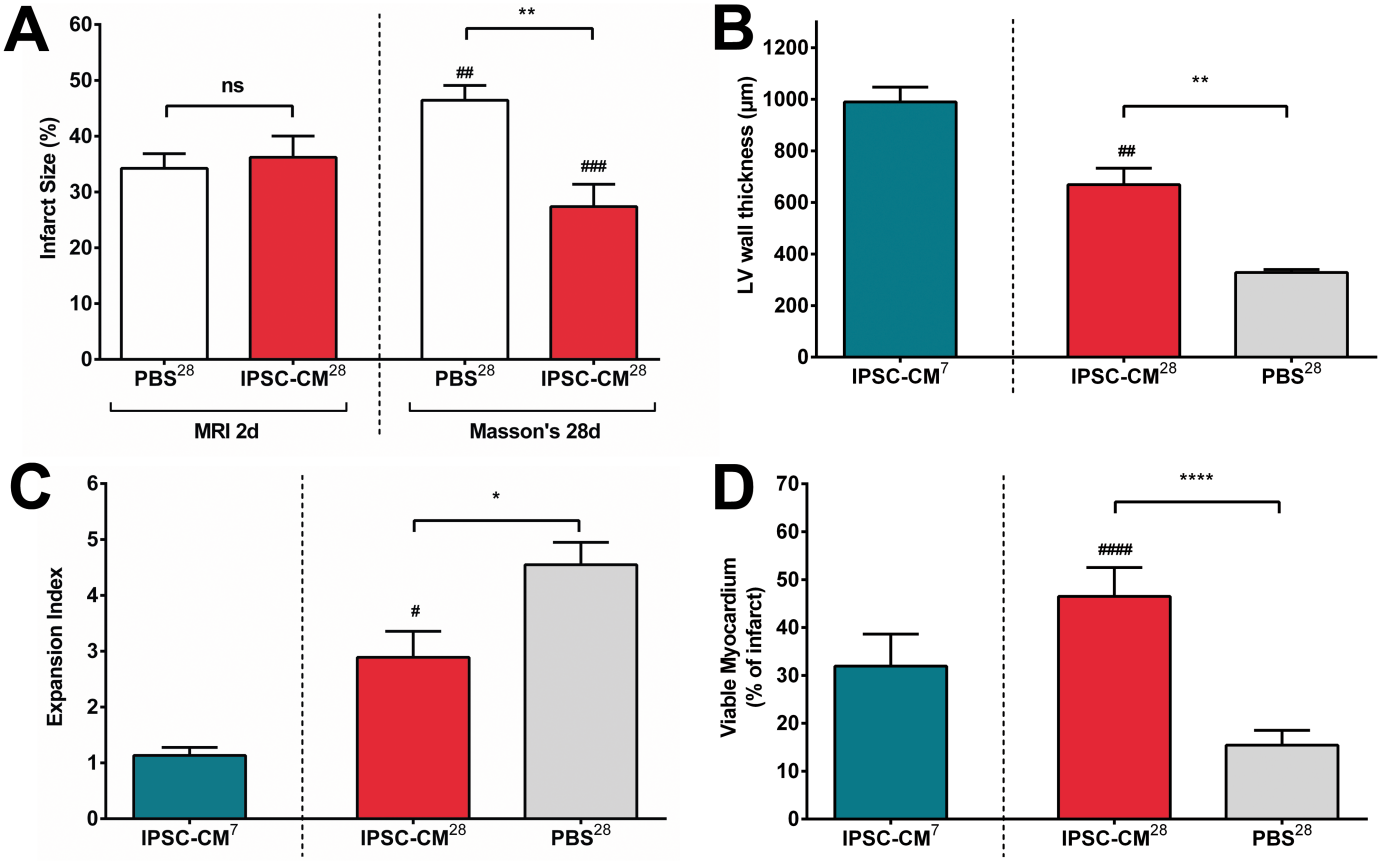
Intramyocardial transplantation of iPSC derived CMs alleviates adverse myocardial remodeling and increases the amount of viable myocardium. **A:** Infarct size (%): after 2 days (MRI): PBS^28^ = 34±3; iPSC-CM^28^ = 36±4; after 28 days (Masson’s): PBS^28^ = 46±3; iPSC-CM^28^ = 25±4 **B:** LV wall thickness after 28 days (Masson’s; μm): iPSC-CM^7^ = 990±57; iPSC-CM^28^ = 669±64; PBS^28^ = 328±12 **C:** Expansion index after 28 days (Masson’s): iPSC-CM^7^ = 1.1±0.1; iPSC-CM^28^ = 2.9±0.5; PBS^28^ = 4.5±0.4 **D:** Viable myocardium (Masson’s; % of infarct area): iPSC-CM^7^ = 32±2; iPSC-CM^28^ = 46±1; PBS^28^ = 15±1. iPSC-CM^28^ vs. PBS^28^: * *P*<0.05, ** *P*<0.01, **** *P*<0.0001. iPSC-CM^7^ vs. iPSC-CM^28^: # *P*<0.05, ##*P*<0.01; ###*P*<0.001; ####*P*<0.0001.

In comparison with infarct sizes determined on day 2 by contrast enhanced MRI, Masson’s Trichrome staining after 28 days showed a significant enlargement of the infarct size in PBS-treated animals. Conversely, infarct size significantly decreased in iPSC-CM-treated animals (Fig 5A). Although animals transplanted with iPSC-CMs did show a remodeling effect including wall thinning, it was significantly less pronounced than in PBS-treated controls (Fig 5B). This was also reflected by the expansion index (EI) that relates LV diameter to LV wall thickness. EI was significantly higher in iPSC-CM^28^ animals than iPSC-CM^7^ animals as a sign for typical post infarct remodeling, but was also significantly larger in PBS^28^ than iPSC-CM^28^ animals (Fig 5C), indicating an improved remodeling in cell treated animals. EI correlated well with infarct size (S8 Fig). These data is in line with the MRI findings and corresponds to a significantly higher amount of viable myocardium (VM) after 28 days in iPSC-CM-treated animals (Fig 5D).

## Discussion

In previous studies we evaluated adult cardiac stem cells [39] und iPSC derived cardiovascular progenitor cells [38] in a murine infarction model. Although these cells were able to form cardiovascular cells *in vitro* and *in vivo* [23,39] an *in vivo* differentiation towards adult CMs was not induced by the host myocardium. Other groups produced similar results [28,29,40]. Since currently there are no methods to sufficiently direct *in vivo* differentiation of immature stem cells, in our opinion a very promising cells source able to form *de novo* contractile myocardium are *bona fide* CMs.

Despite recent progress in hCM differentiation [41], further efficient enrichment of pluripotent stem cell (PSC)-derived CMs appears still necessary in view of the risk of teratoma formation. To purify authentic CMs from PSCs, various methods have been proposed including genetic approaches [32,33]. Although genetic enrichment has so far been considered as barely clinically applicable this view has now changed due to recent groundbreaking developments in targeted genome engineering. Besides ZFN (zinc finger nuclease) and CRISPR (clustered regularly interspaced short palindromic repeats)/Cas9 technologies, especially TALEN (transcription activator-like effector nuclease)-based gene targeting represents a highly efficient method for introduction of transgenes into safe harbor sites such as adeno-associated virus integration site 1 (AAVS1) [42]. Notably, this approach can be regarded as much safer than the common random integration of transgenes and provides well controllable expression levels in undifferentiated iPSCs as well as their differentiated derivatives. Different groups were able to enrich CMs from differentiating transgene PSC clones, mostly from ESCs [43]. Van Laake et al. reported the purification of CMs from transgenic iPSCs based on a NKX2.5-GFP reporter system and a directed differentiation approach. FACS sorted, dissociated cardiovascular iPSC derived progenitor cells were transplanted into the infarcted myocardium of NOD scid mice. Small grafts were detected after two weeks but did not show a clear mature CM phenotype [28]. Ma et al. reported an antiobiotic selection based method to purify CMs from human iPSCs. These CMs had similar electrophysiological properties compared to human cardiac myocytes [44].

We used a genetically engineered iPSC clone especially developed for maocardial regeneration [35]. Similar to the pioneering work in ESCs from Klug et al. [45] the iPSC clone carries an antibiotic resistance gene that is expressed under control of a cardiac-specific promoter. We were able to obtain contracting cardiac bodies of almost pure CMs. CMs showed a clear mature phenotype and developed into an atrial and ventricular phenotype. The ratio of nearly 1:1 is well in line with the electrophysiology data obtained by others [44]. Regarding a potential contamination with residual undifferentiated iPSCs we could show, that the Oct3/4 dependent GFP expression of the transgenic iPSC clone disappeared during differentiation. In this context, pureness of yielded CMs is very important since residuals of undifferentiated iPSCs implies potential teratoma formation once transplanted [46]. Moreover, iPSC derived CMs showed a low proliferation potential as determined by Ki67 staining. Expectedly, *in vivo* grafts did not show mitotic activity after 7 days. We refrained from additional genetic manipulation of the transgenic iPSC clone to enable a reporter gene based identification as described by our group before [38]. Using a vitality sensitive intracellular fluorescence tracer (CFDA SE) we were able to effectively label iPSC derived CBs for *in vivo* identification. Our data show that iPSC derived CMs were viable after antibiotic selection process within the three-dimensional CB environment of almost pure CMs. The fluorophore remained visible even in apoptotic CM grafts *in vivo* over a period of 28 days. Moreover, resulting CM preparations were capable to form contractile myocardial tissue *in vitro*, based on non-dissociated CM aggregates called cardiac bodies (CBs). After intramyocardial injection into ischemic myocardium, iPSC derived CMs exhibited an authentic adult CM appearance despite their original spherical organisation within CBs. We believe that their alignment and longitudinal organisation is induced by the directed strain within in the host’s myocardium. This finding has also been described for BCTs and indicates a viable CM response after transplantation [35]. This self-organisation as a mature CM syncytium in our opinion also results from the use of non-dissociated CBs rather than dissociated single CMs. Although we were able to establish a Connexin 40 and 45 expression within CBs, coupling with the host myocardium was not observed *in vivo*. In our model, CMs were indirectly connected to the host myocardium after injection into the infarct area because of a surrounding cellular infiltration after 7 days and scar formation after 28 days. CM graft appearance changed over a period of 28 days towards an unorganized morphology and reduced size indicating late cell death. Impaired CM viability in models of myocardial infarction has been frequently described and attributed to the cytotoxic environment after myocardial infarction or dissociation of CMs [34,35,47]. Our histological data suggests that even after transplantation of non-dissociated CBs disruption of graft tissue due to a weak cell-cell connection could be another reason for delayed CM apoptosis and impaired connection to the host myocardium. This may result from the almost pure CM composition of CBs. Accordingly, Kensah at al. described that the lack of fibroblasts within BCTs obtained from iPSC derived CBs led to an incomplete extracellular matrix remodelling and CB fusion. The addition of fibroblasts resulted in an improved structure and function of BCTs [35]. Future experiments will have to establish, whether the addition of fibroblasts can ensure graft survival and may improve the myocardial connection in our model.

## Conclusions

We could show that direct intramyocardial transplantation of iPSC derived CMs as three-dimensional CBs results in a significant functional improvement and attenuated adverse remodelling 28 days after acute myocardial infarction as determined by a combination of MRI, pressure-volume loop analysis and histological morphology assessment. LV thickness in the infarct zone was preserved despite decreasing iPSC-CM graft size thereby preventing progressive LV dilatation. In contrast to former studies by our group using adult cardiac stem cells [39] and Flk-1^pos^ iPSC derived progenitor cells [38] in the same model, we were also able to show significant improvement of LV contractility parameters based on PV loop assessment. Further studies providing mechanical insights regarding the interaction between host myocardium and transplanted cells would undoubtedly contribute to improve myocardial stem cell therapy.

## Supporting information

S1 Fig eGFP expression. Undifferentiated IPSCs showed a marked Oct3/4-mediated eGFP expression and a high proportion of mitotically active Ki-67 positive cells. Most cells were positive for both markers. Few cells were Oct3/4-eGFP negative and Ki-67 positive (arrowheads). Rarely cells were Oct3/4-eGFP positive and Ki-67 negative (*). Scale bar: 50μm.
(TIF)

S2 Fig Ki-67 expression I. Differentiated CBs at dd14 lost the intrinsic Oct3/4-mediated eGFP signal compared to undifferentiated IPSCs (S1 Fig) and were predominantly negative for nuclear Ki67. Scale bar: 100μm.
(TIF)

S3 Fig Ki-67 expression II. Reseeded IPSC-CMs showed a low proportion of Ki-67 positive cells (4.3±3.0%, N = 7). Scale bar: 100μm.
(TIF)

S4 Fig Connexin 45 expression. Differentiated CBs on dd14 were positive for Connexin 45. Scale bar: 100μm.
(TIF)

S5 Fig Connexin 40 expression. Differentiated CBs on dd14 were positive for Connexin 40. Scale bar: 100μm.
(TIF)

S6 Fig Overall mortality. Differences between non-infarcted animals (Sham^28^) and infarcted animals (PBS^28^; IPSC-CM^7^, IPSC-CM^28^) were statistically not significant. (Sham^28^ vs. PBS^28^: *P* = 0.061; Sham^28^ vs. IPSC-CM^7^: *P* = 0.26; Sham^28^ vs. IPSC-CM^28^: *P* = 0.16; PBS^28^ vs. IPSC-CM^7^: *P* = 0.70; PBS^28^ vs. IPSC-CM^28^: *P* = 0.72; IPSC-CM^7^ vs. IPSC-CM^28^: *P* = 1.00) Most deceased animals died perioperatively. Hence, mortality within the 7 day group (IPSC-CM^7^) was similar to 28 day myocardial infarction groups (PBS^28^, IPSC-CM^28^).
(TIF)

S7 Fig MRI (POD 27) findings correlated well with CC measurements (POD 28). **A:** MRI LV-EF (%) vs. CC LV-EF (%). **B:** MRI EDV (μl) vs. CC EDV (μl). Volume values were typically underestimated by CC evaluation.
(TIF)

S8 Fig Morphometry (Masson’s Trichrome Staining, POD28). Expansion Index (EI) correlated well with Infarct Size.
(TIF)

S1 File Supporting file. (DOCX)

S1 Video IPSC-derived “Cardiac bodies” (CBs) after antibiotic cardiomyocyte (CM) selection on differentiation day 14. (MPG)

S2 Video IPSC-derived “Cardiac bodies” (CBs) after intracellular Vybrant ^®^ CFDA SE (carboxy-fluorescein diacetate succinimidyl ester) tracer staining. CBs remain vital and contracting.
(MPG)
