# Supplementary figures and images for: Transplantation of purified iPSC-derived cardiomyocytes in myocardial infarction
Source: PLoS One. 2017 May 11;12(5):e0173222. doi: 10.1371/journal.pone.0173222 (PMC5426598; doi:10.1371/journal.pone.0173222)

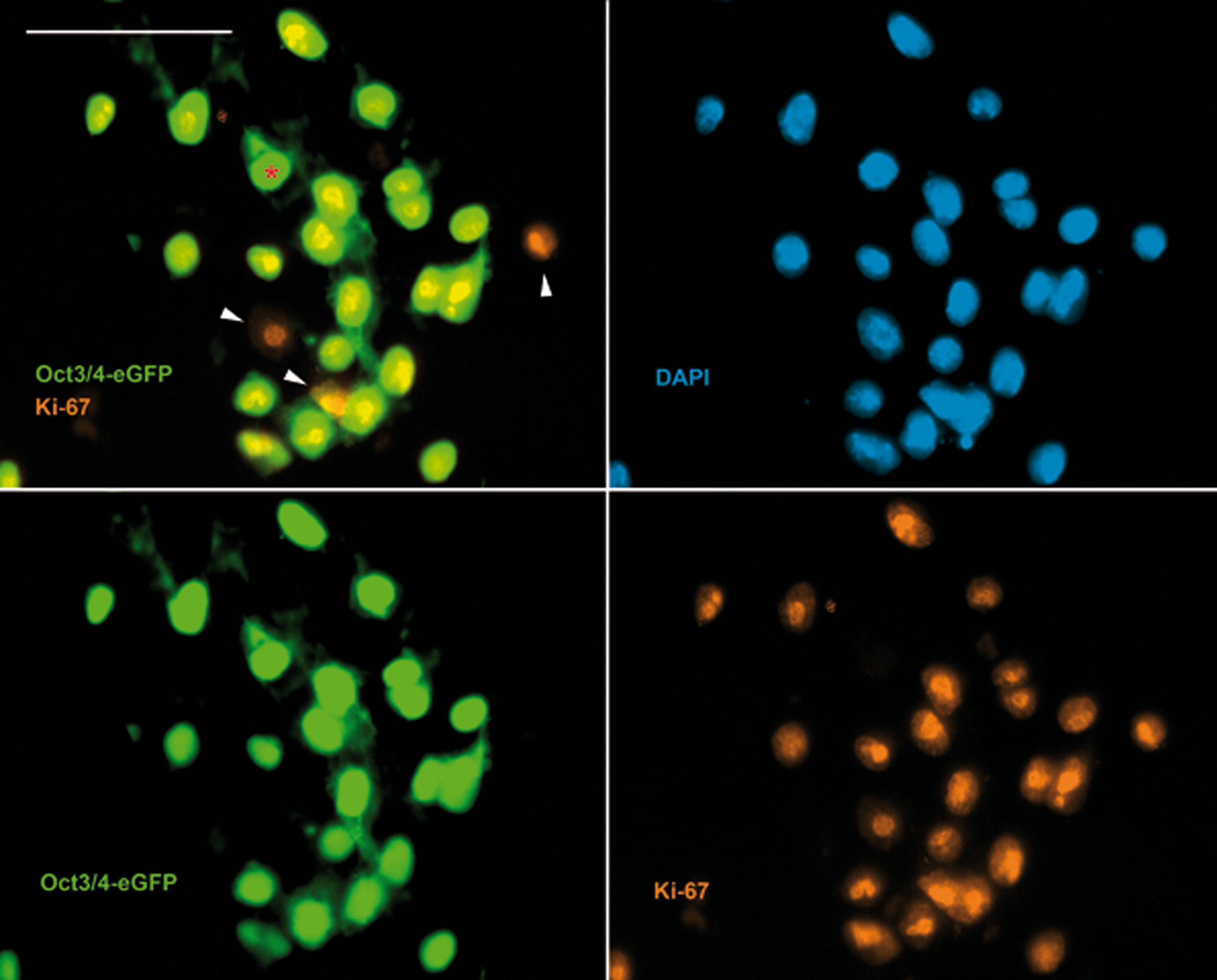

Supplement: S1 Fig — Undifferentiated IPSCs showed a marked Oct3/4-mediated eGFP expression and a high proportion of mitotically active Ki-67 positive cells. Most cells were positive for both markers. Few cells were Oct3/4-eGFP negative and Ki-67 positive (arrowheads). Rarely cells were Oct3/4-eGFP positive and Ki-67 negative (*). Scale bar: 50μm. (TIF) [file pone.0173222.s001.tif]

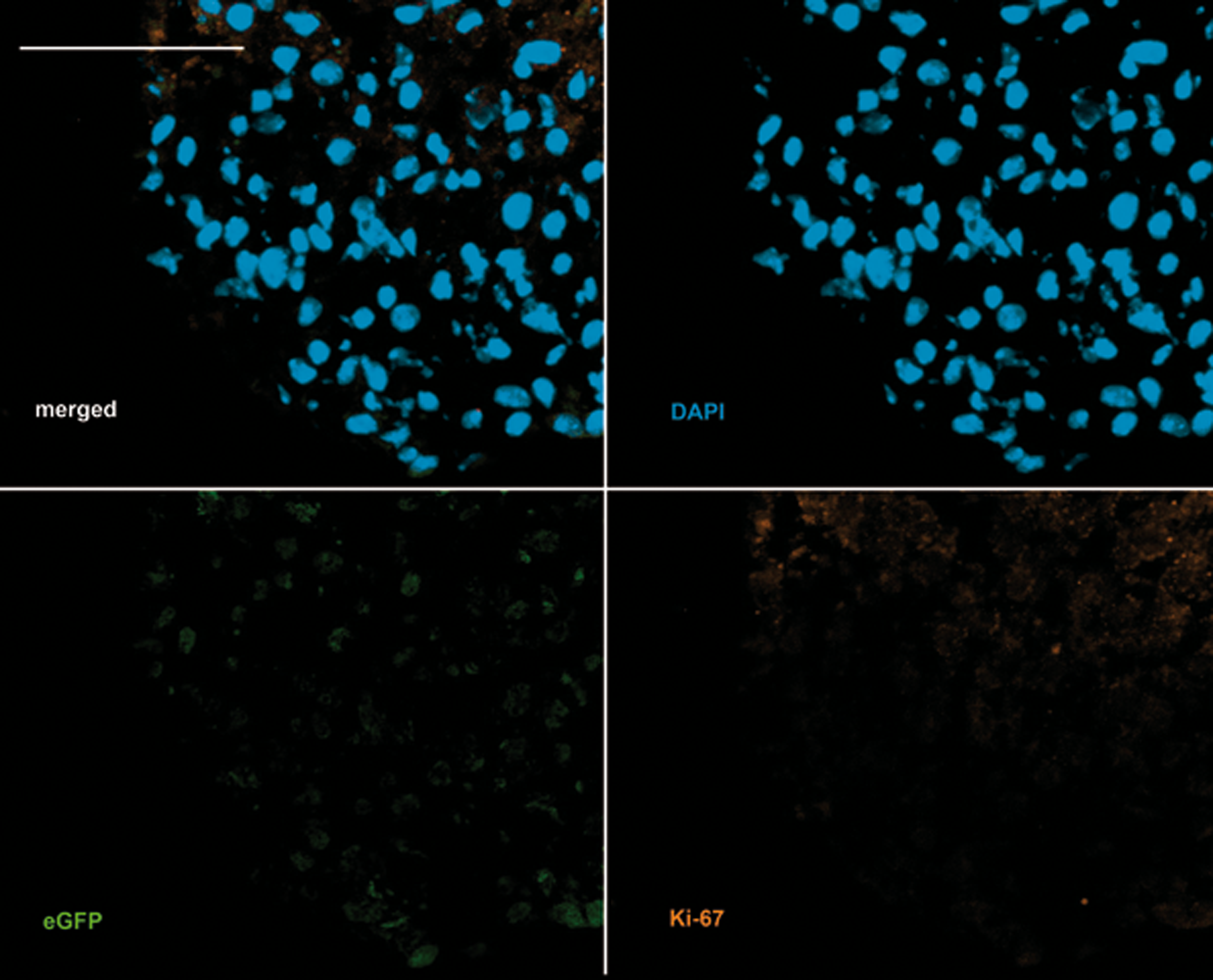

Supplement: S2 Fig — Differentiated CBs at dd14 lost the intrinsic Oct3/4-mediated eGFP signal compared to undifferentiated IPSCs (S1 Fig) and were predominantly negative for nuclear Ki67. Scale bar: 100μm. (TIF) [file pone.0173222.s002.tif]

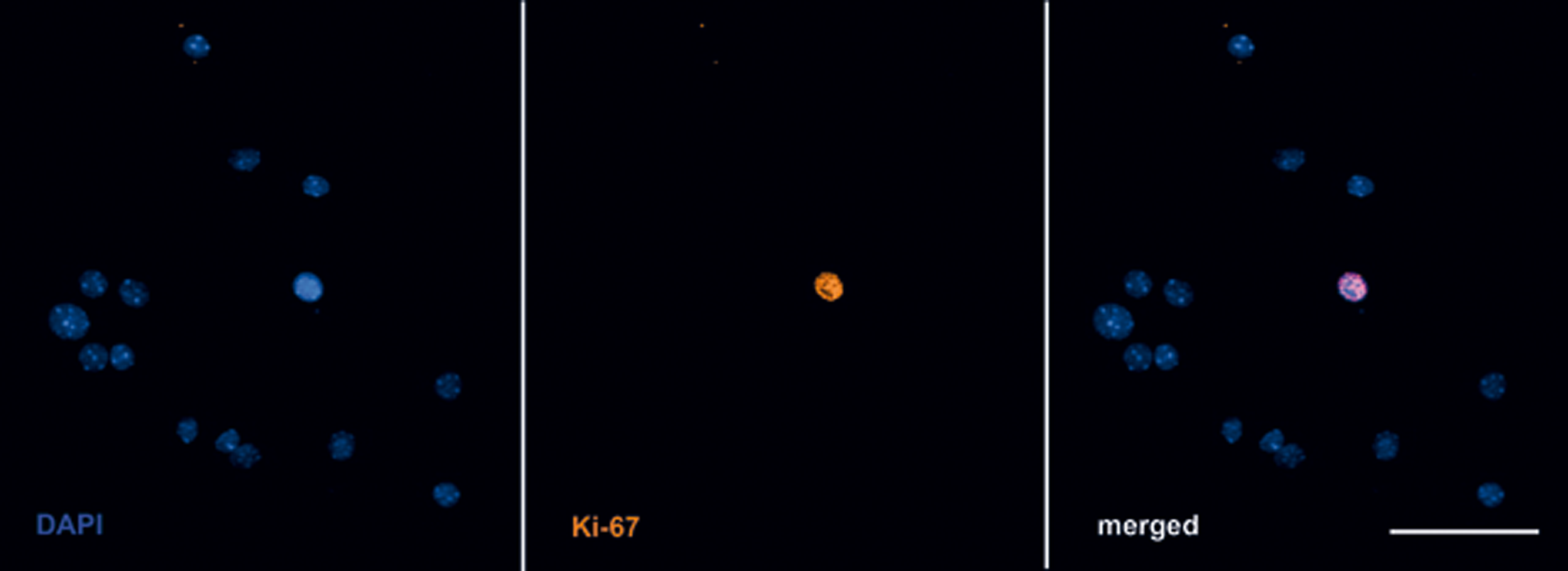

Supplement: S3 Fig — Reseeded IPSC-CMs showed a low proportion of Ki-67 positive cells (4.3±3.0%, N = 7). Scale bar: 100μm. (TIF) [file pone.0173222.s003.tif]

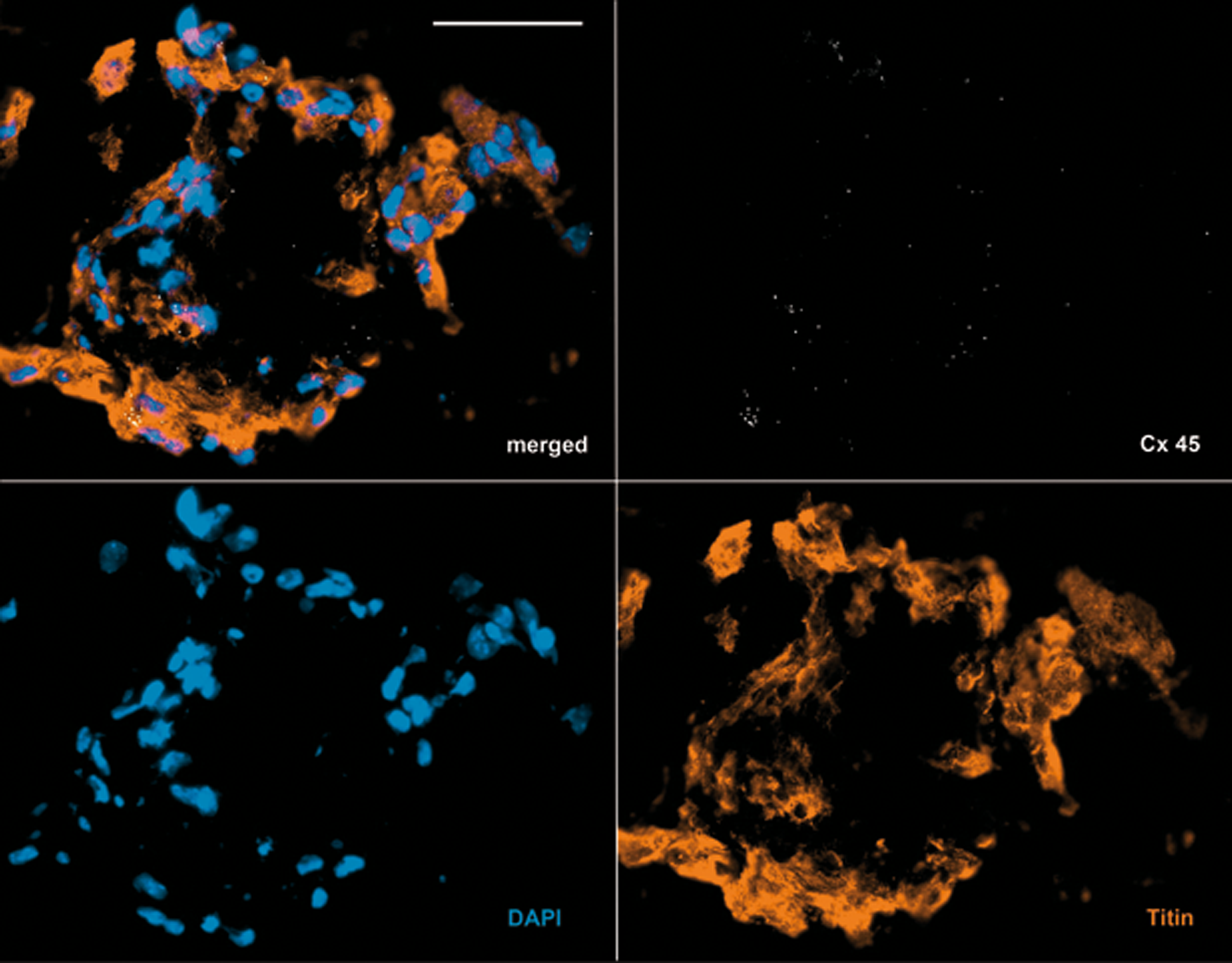

Supplement: S4 Fig — Differentiated CBs on dd14 were positive for Connexin 45. Scale bar: 100μm. (TIF) [file pone.0173222.s004.tif]

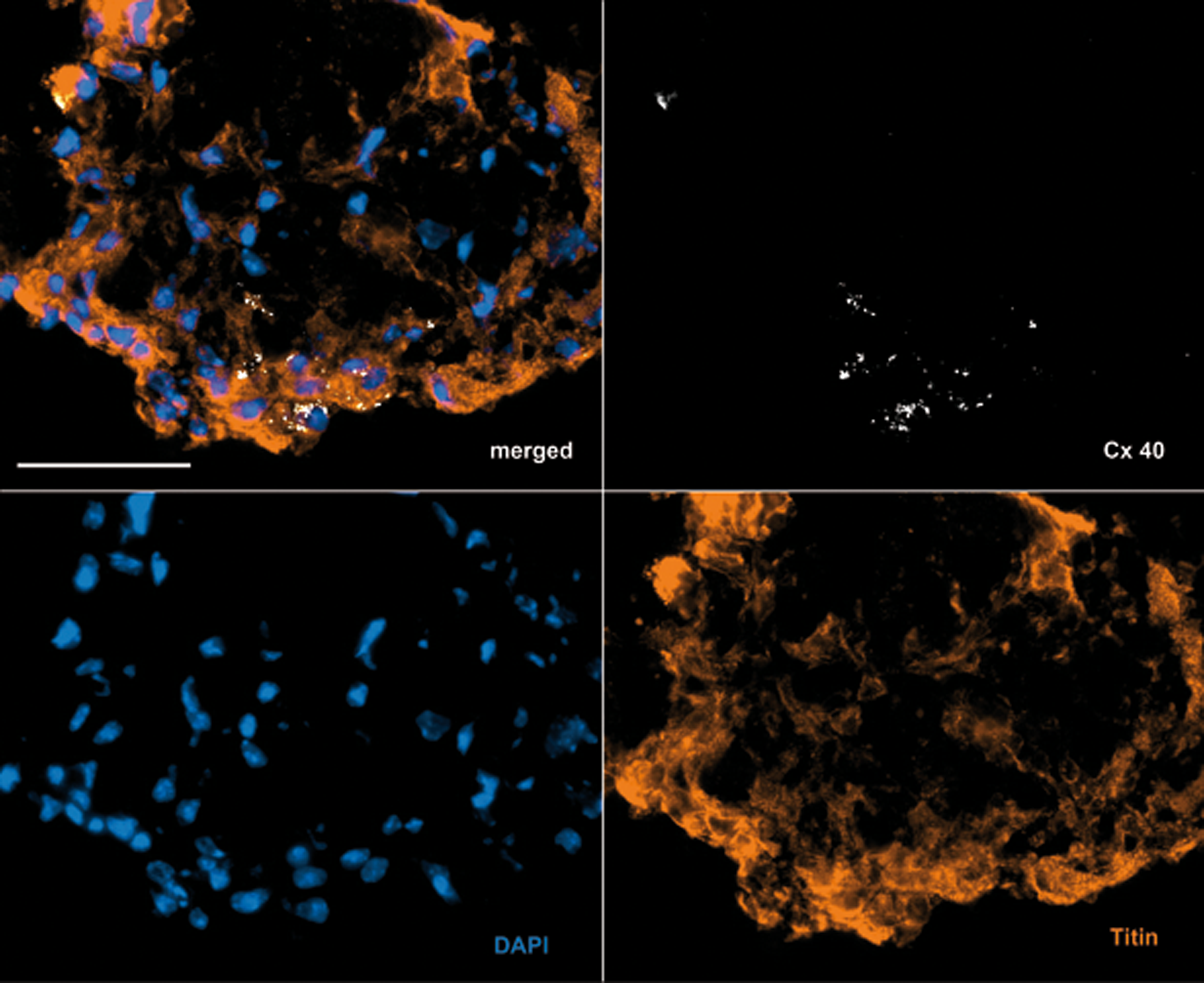

Supplement: S5 Fig — Differentiated CBs on dd14 were positive for Connexin 40. Scale bar: 100μm. (TIF) [file pone.0173222.s005.tif]

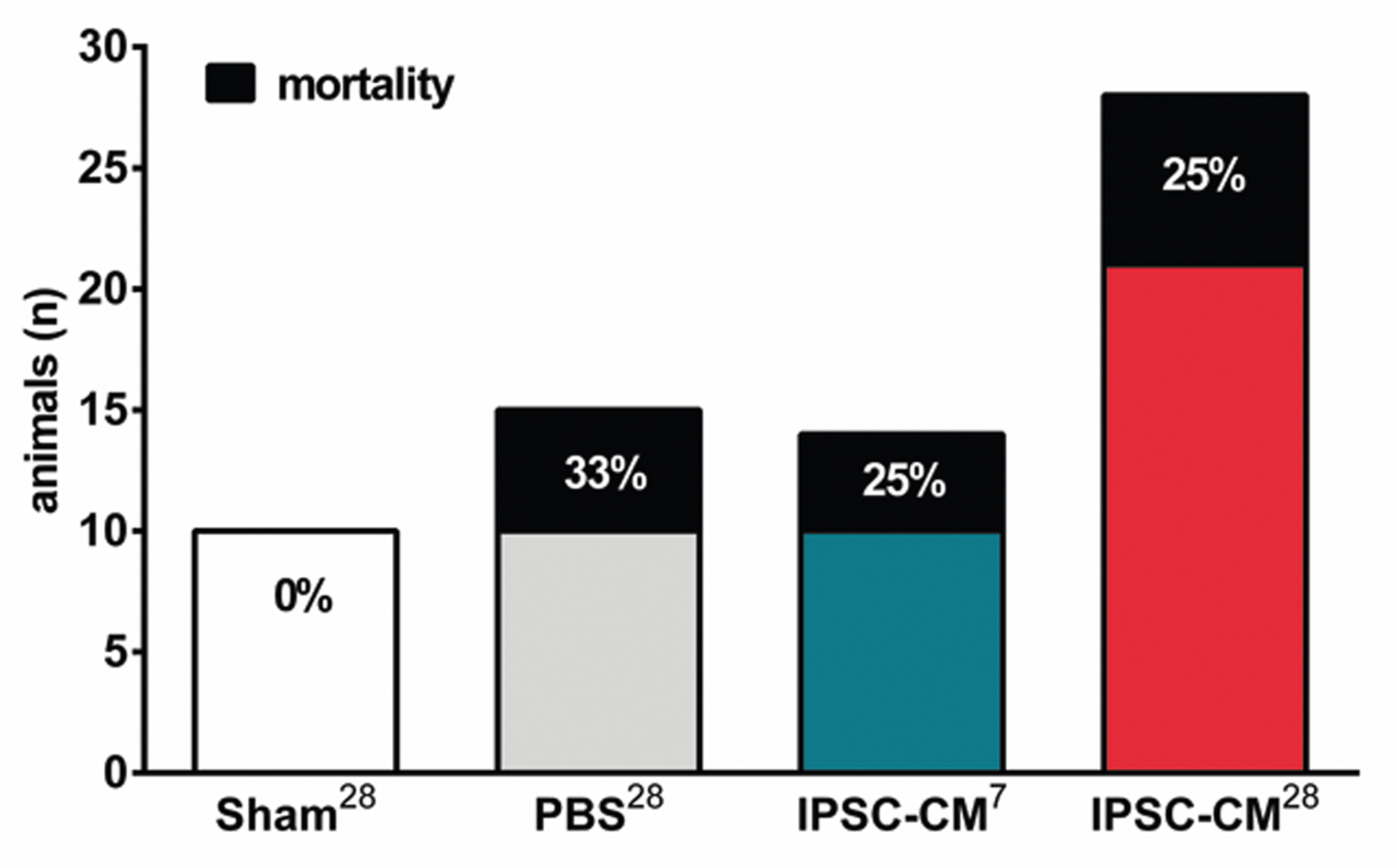

Supplement: S6 Fig — Differences between non-infarcted animals (Sham28) and infarcted animals (PBS28; IPSC-CM7, IPSC-CM28) were statistically not significant. (Sham28 vs. PBS28: P = 0.061; Sham28 vs. IPSC-CM7: P = 0.26; Sham28 vs. IPSC-CM28: P = 0.16; PBS28 vs. IPSC-CM7: P = 0.70; PBS28 vs. IPSC-CM28: P = 0.72; IPSC-CM7 vs. IPSC-CM28: P = 1.00) Most deceased animals died perioperatively. Hence, mortality within the 7 day group (IPSC-CM7) was similar to 28 day myocardial infarction groups (PBS28, IPSC-CM28). (TIF) [file pone.0173222.s006.tif]

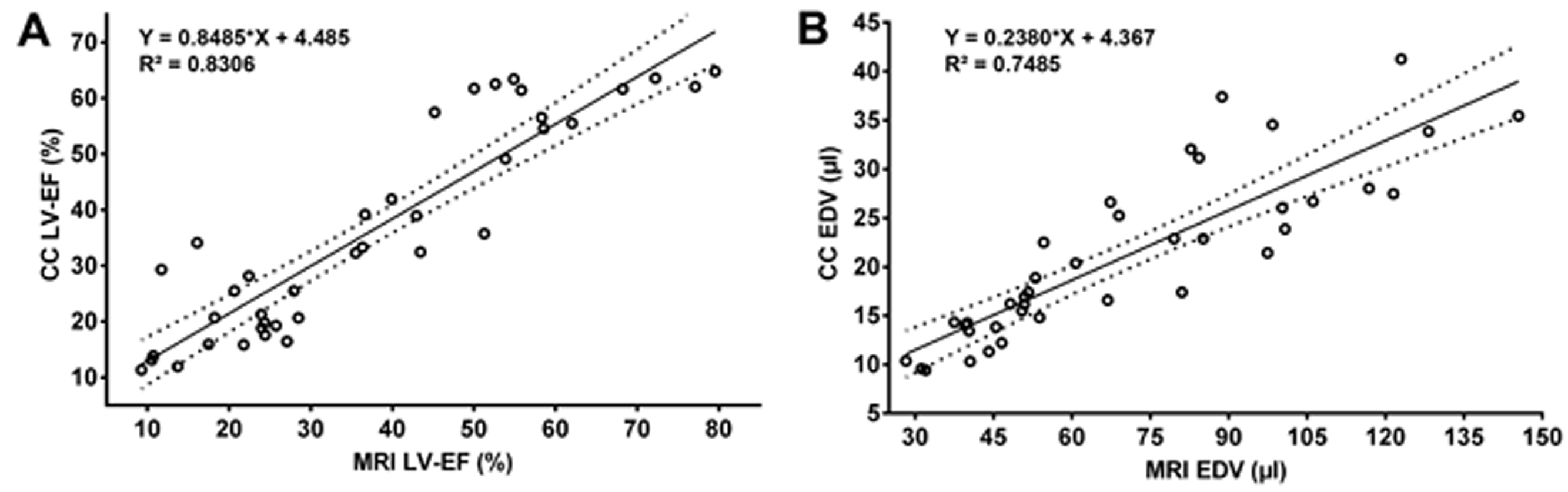

Supplement: S7 Fig — A: MRI LV-EF (%) vs. CC LV-EF (%). B: MRI EDV (μl) vs. CC EDV (μl). Volume values were typically underestimated by CC evaluation. (TIF) [file pone.0173222.s007.tif]

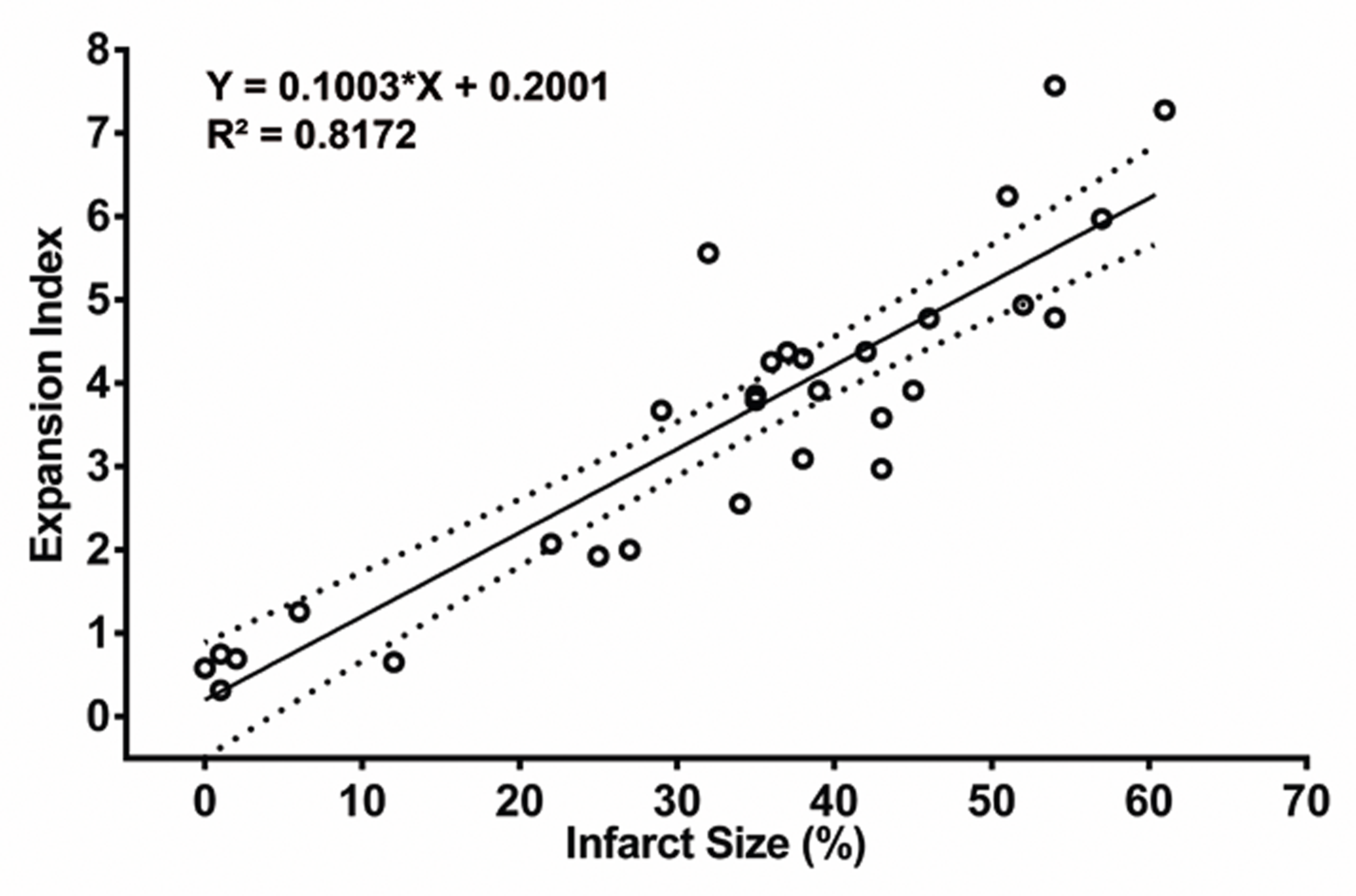

Supplement: S8 Fig — Expansion Index (EI) correlated well with Infarct Size. (TIF) [file pone.0173222.s008.tif]
