# Supplementary material for: Transplantation of purified iPSC-derived cardiomyocytes in myocardial infarction
Source: PLoS One. 2017 May 11;12(5):e0173222. doi: 10.1371/journal.pone.0173222 (PMC5426598; doi:10.1371/journal.pone.0173222)
Supplement: S1 File — (DOCX) [file pone.0173222.s009.docx]

**Supplemental Methods**

**IPSC Culture Protocols and Antibiotic Selection**

IPSCs were cultured as established and described by Kensah et al. recently [35]. In summary undifferentiated murine IPSCs were cultured on a mitotically inactivated feeder layer. Cells were passaged every 3 to 4 days after detaching the colonies with collagenase IV (0,2% w/v) and dissociation into single cells using TrypLE Select (both Life Technologies).

The establishment of a transgenic murine IPSC clone for antibiotic selection has been described in detail [35]. Semi-quantitative RT-PCR confirmed stable resistance gene (Zeo^R^) expression in the transfected clone on day 10 of cardiac differentiation. The clone was used for all subsequent transplantation studies.

Cardiac differentiation was initiated using a dynamic suspension culture protocol. Initially cardiac differentiation was induced by the hanging drop (HD) technique (1 x10³ undifferentiated murine IPSCs / droplet in 33 µL differentiation medium, 160 HD/dish, 60 dishes). On differentiation day (dd) 3 embryoid bodies (EBs) were transferred to 150 mm petri dishes (Sarstedt, Nuembrecht, Germany) at a density of 1600 EBs / 20 mL differentiation medium. Cultures were supplemented with 100 µM L-ascorbic acid-2-phosphate (Sigma-Aldrich, Hamburg, Germany) from dd3 to dd7. Dishes were placed on an orbital shaker at 70 rpm.

Cardiac differentiation efficiency was determined by transferring EBs to a 96 well plate and subsequent microscopic assessment. The amount of persisting undifferentiated murine IPSCs was determined by their intrinsic expression of eGFP under control of the Oct3/4-promotor.

Antibiotic selection was performed from dd7 to dd14. Differentiation medium was therefore supplemented with 400 µg/mL Zeocin® from dd7 on. Medium was changed on dd10. On dd14 cells were processed for intramyocardial transplantation based on the finding of Kensah et al. [35] that an optimal combination of cell yield and vitality was found between dd13 and dd14.

For immunohistological evaluation of single cells CBs were dissociated with 0,1% (w/v) collagenase B and Accutase^TM^. Cells were seeded onto 24 well plates coated with 5 µg/mL fibronectin and grown for additional three days in differentiation medium without antibiotic selection.

**Determination of Average IPSC-CM Cell Count**

Non-dissociated CBs were used for transplantation purposes to maximize CM vitality and to preserve cell-cell contacts and functional integrity of CBs. Using the above mentioned protocol Kensah et al. [35] have determined a cell count of 1678 ±191 IPSC derived CMs per CB.

For cell count analysis, 800 CBs from different (n=5) IPSC differentiation experiments were processed. Cells were dissociated using collagenase B and Accutase^TM^. Single cells were counted with a CASY system to determine total cell count and number of viable cells. Average total cell count was 3,22 x 10^6^ cells per 800 CBs, proportion of viable cells after dissociation was 72%. These findings are in line with the data of Kensah et al. [35]. For each animal, 9600 HD were prepared. The total amount of resulting CBs was utilized for transplantation.

**Cell Processing for Intramyocardial Transplantation**

To enable easy and fast histological detection, CBs were labeled with a vitality sensitive fluorescence cell tracer (Vybrant® CFDA SE Cell Tracer Kit, LifeTechnologies^TM^, Darmstadt, Germany) according to the manufacturer’s protocol. CFDA SE (carboxy-fluorescein diacetate succinimidyl ester) passively diffuses into cells and is colorless until its acetate groups are cleaved by intracellular esterases. This yields the fluorescent dye-protein, which binds with intracellular amines and forms fluorescent conjugates. Unconjugated reagent passively diffuses to the extracellular medium and can be washed away. Approximate fluorescence excitation/emission maxima are 492/517 nm. The detailed protocol and additional information can be retrieved at www.invitrogen.com.

For fluorescence staining CBs were washed with PBS, incubated with 10 µM CFDA SE for 15 min at 37°C and thereafter washed with differentiation medium. CBs were then incubated for 30 min at 37°C to ensure complete conversion of the fluorescence probe.

Fluorescence and CB appearance was microscopically assessed to ensure successful staining of viable and contracting CBs. Only then, cells were considered for transplantation. CBs were then centrifuged and the supernatant was discarded.

Cells were immediately transferred to the animal lab for intramyocardial transplantation into LAD ligated mice. Transplantation was performed within two hours after cell processing.

**Myocardial Infarction Model**

Myocardial infarction was induced as previously described [35]. In short, immunodeficient SCID beige mice (15-21 g, 8-11 weeks, Charles River, Germany) were anaesthetized with isoflurane (2%), orally intubated and mechanically ventilated (MiniVent, Hugo Sachs Elektronik, March-Hugstetten, Germany). Anaesthesia was maintained using isoflurane (1.0-1.5%). A left anterior thoracotomy was performed and the left anterior descending artery (LAD) was permanently ligated using an 8/0 Prolene suture (Ethicon, Johnson&Johnson Medical, Norderstedt, Germany). Myocardial ischemia was verified by intraoperative ECG (FMI/MIL MPVS-Ultra ECG cable, FMI, Seeheim-Ober Beerbach, Germany). Cell or placebo injections (PBS) were performed using a tuberculine syringe (BD) with a 30G (0.30mm) needle.

**Magnetic Resonance Imaging (MRI)**

MRI studies were conducted as previously described [38]. We used a 7 Tesla (300 MHz) scanning system (PharmaScan, Bruker, Etlingen, Germany) with a 160-mm horizontal bore. A 38 mm resonator coil (Bruker) was used to transmit and receive signals.

Animal body temperature was maintained at 37°C using a water-perfused heating mat. Initially mice were anaesthetized with 3% isoflurane for 3 min and placed in a coil with a pneumatic pillow for respiration monitoring and maintained at 1.5–2% isoflurane. ECG electrodes were attached to the left fore and right hind limb. ECG- and respiration-triggered image acquisition as well as retrospective triggered imaging (Intragate®) was performed using the Bruker Paravision 5.0 software.

On postoperative day 2 (POD 2), infarct size was determined by contrast enhanced magnetic resonance imaging (MRI). The contrast agent gadolinium-DOTA (Dotarem, Guerbet Gorinchem, Netherlands) was injected via the tail vein. To cover the left ventricle (LV), a multi-slice RARE sequence (14 contiguous, 0.6mm slices, TR 1500, TE 9ms, FA180 deg, AVG 3) was used. Late enhancement was quantified as percentage of the left ventricular mass using the “Mass 4Mice” analysis software (Medis Medical Imaging Systems, Netherlands).

On POD 27, animals were evaluated for cardiac function. We performed single-slice Intragate FLASH cine imaging using 6-9 contiguous 1-mm thick slices covering the LV to analyze function. Per cardiac cycle, 20 frames were acquired. The Intragate cine FLASH sequences used a 10 deg flip angle, 8 ms repetition time, 3.6 ms echo time, and 300 repetitions. The field of view was 40x40 mm^2^, projected on a 256x256 matrix resulting in 0.0156 cm/pixel. Left ventricular volumetric parameters and ejection fraction (LV-EF) were measured and calculated with the “Mass 4Mice” analysis software (Medis Medical Imaging Systems).

**Conductance catheter (CC) analysis**

Pressure volume loop evaluation was performed according to previous studies [38,39]. On POD 28, animals were anaesthetized with isoflurane (2%), orally intubated, mechanically ventilated and placed supine on a heating plate. Anaesthesia was maintained using isoflurane (1.0-1.5%). A longitudinal cervical incision was performed to expose the trachea and cervical vessels. A 1.0 F conductance catheter (MIL-PVR-1030, Millar Instruments, FMI, Seeheim, Germany) with an electrode spacing of 3 mm was positioned into the left ventricle via the right carotid artery. The left external jugular vein was cannulated for hypertonic saline injection to obtain parallel conductance of the left ventricular myocardium. Volumetric calibration for slope factor α was performed via a calibration cuvette (Mo.910-1049, Millar Instruments). Data were obtained using a Millar transducer system (MPVS-Ultra, Millar Instruments) and PowerLab data acquisition platform (ADInstruments, FMI). Pressure-volume signals were recorded in steady state to acquire volumetric and functional parameters. Preload independent functional parameters were determined from pressure volume loop relations during load reduction via temporary occlusion of the inferior vena cava after abdominal incision. Following the operation, animals were sacrificed for histological evaluation.

**Cryosectioning and (Immuno)-Staining**

Hearts were perfused with PBS, explanted and fixed in 4% paraformaldehyde (PFA) followed by overnight cryopreservation in 30% sucrose (Sigma-Aldrich). Hearts were embedded in TissueTek (Sakura Finetek, Heppenheim, Germany) and stored at -80°C.

Subsequently, starting at the atrio-ventricular transition a series of fourteen 8 μm cryosections was produced (112 µm). The following 192 µm were discarded. This resulted in a distance between each section series of 304 µm. The procedure was repeated until the entire heart was cut. Masson’s Trichrome staining was performed on the first section of each series, the other slices were used for immunostaining. Cryosections were mounted on object slides and air-dried overnight.

CBs were centrifuged, fixed in 4% paraformaldehyde (PFA) and gradually frozen in TissueTek (Sakura) at -80°C. Cryosections were obtained at 8 µm intervals, mounted on object slides and air-dried overnight.

Cryosections were fixed with 2-4% PFA for 4 min at 4°C and subsequently blocked with 5% donkey serum and 0.25% Triton X-100 (Sigma-Aldrich) diluted in Tris-buffered saline for 20 min at room temperature (RT). Sections were stained with primary and secondary antibodies according to standardized protocols. Corresponding isotype antibodies were used for negative controls. Antibodies used in these studies are summarized in Table A. Nuclei were stained with DAPI. For immuno-fluorescence microscopy, we used an Axio Observer A1 fluorescence microscope (Zeiss, Oberkochen, Germany) and the Axiovision Software 4.71 (Zeiss).

Masson Goldner Trichrome staining was performed using the Roth staining kit (Carl Roth GmbH, Karlsruhe, Germany) according to the manufacturer’s protocol. Sections were fixed with acetone at -20°C, stained with Weigert’s iron hematoxylin solution for 3 min and rinsed with water for 13 min. Specimen were then incubated with Goldner’s solution I (Fuchsin, 10min), rinsed with 1% acetic acid (30 sec), incubated with Goldner’s solution II (Orange G, 3min), rinsed with 1% acetic acid (30 sec), incubated with Goldner’s solution III (Light green SF, 21sec) and rinsed with 1% acetic acid (3 min). Thereafter, sections were dehydrated by ascending alcohol series (3-5 sec, 70-96% alcohol) and treated with Xylol I and II solution.

**Table A. Antibodies used for Immunostaining.**

**A: Primary antibodies**

| **Species** | **Class** | **Name** | **Label** | **Vendor** | **Dilution** |
| --- | --- | --- | --- | --- | --- |
| mouse | IgG1 | Anti-cardiac troponin T | - | Thermo Scientific, Dreeich, Germany | 1:100 |
| mouse | IgG1 | Isotype control antibodies | - | DakoCytomation, Glostrup, Denmark | 1:50 |
| mouse | IgM | Anti-Titin | - | Hybridoma Banks, University of Iowa | 1:30 |
| mouse | IgM | Isotype control antibodies | - | DakoCytomation, Glostrup, Denmark | 1:85 |
| mouse | IgG1 | Anti-α-actinin | - | Sigma-Aldrich, Hamburg, Germany | 1:800 |
| mouse | IgG1 | Isotype control antibodies | - | DakoCytomation, Glostrup, Denmark | 1:6 |
| mouse | IgG2a | Anti-Myosin Light Chain 2v | - | Synaptic Systems, Goettingen, Germany | 1:200 |
| mouse | IgG2a | Isotype control antibodies | - | DakoCytomation, Glostrup, Denmark | 1:20 |
| mouse | IgG2b | Anti-Myosin Light Chain 2a | - | Synaptic Systems, Goettingen, Germany | 1:200 |
| mouse | IgG2b | Isotype control antibodies | - | DakoCytomation, Glostrup, Denmark | 1:20 |
| rat | IgG2a | Anti-Ki-67 | - | DakoCytomation, Glostrup, Denmark | 1:40 |
| rat | IgG2a | Isotype control antibodies | - | R&D Systems, Minneapolis, USA | 1:40 |
| goat | IgG | Anti-Connexin 40 | - | Santa Cruz Biotechnology, Heidelberg, Germany | 1:100 |
| goat | IgG | Isotype control antibodies | - | Santa Cruz Biotechnology, Heidelberg, Germany | 1:100 |
| goat | IgG | Anti-Connexin 45 | - | Santa Cruz Biotechnology, Heidelberg, Germany | 1:100 |
| goat | IgG | Isotype control antibodies | - | Santa Cruz Biotechnology, Heidelberg, Germany | 1:100 |
|  |  |  |  |  |  |

**B: Secondary Antibodies**

| **Species** | **Class** | **Name** | **Label** | **Vendor** | **Dilution** |
| --- | --- | --- | --- | --- | --- |
| donkey | - | Anti-goat IgG | Dylight 649 | Dianova, Hamburg, Germany | 1:300 |
| donkey | - | Anti-mouse IgM | Dylight 549 | Dianova, Hamburg, Germany | 1:300 |
| donkey | - | Anti-rat IgG | Cy3 | Dianova, Hamburg, Germany | 1:200 |
| goat | - | Anti-mouse IgG | Dylight 549 | Dianova, Hamburg, Germany | 1:300 |
| goat | - | Anti-mouse IgM | Dylight 649 | Dianova, Hamburg, Germany | 1:300 |

**Morphometry**

Infarct size was determined on Masson Goldner Trichrome stained sections at 304 µm intervals by calculating the ratio of the circumferential infarct length to the circumferential length of healthy LV myocardium as described previously [33]. To determine ventricular dilatation and wall thinning minimal left ventricular wall thickness (LV-WT), the relative area of viable myocardium (VM) and the expansion index (EI; [endocardial circumference/epicardial circumference]x[posterior wall thickness/anterior wall thickness]) were measured according to Tang et al. Data were obtained by computer-assisted morphometry (Image J 1.40g, NIH, USA).

IPSC-CM graft size was determined using a pixel-based method (Image J 1.46r, NIH, USA). Every section containing grafted cells was evaluated. Graft size (µl) was calculated as the average graft area per section multiplied by section length.
